# Epigallocatechin-3-gallate (EGCG) Alters Histone Acetylation and Methylation and Impacts Chromatin Architecture Profile in Human Endothelial Cells

**DOI:** 10.3390/molecules25102326

**Published:** 2020-05-16

**Authors:** Oskar Ciesielski, Marta Biesiekierska, Aneta Balcerczyk

**Affiliations:** 1 Department of Molecular Biophysics, Faculty of Biology and Environmental Protection, University of Lodz, Pomorska 141/143, 90-236 Lodz, Poland; oskar.ciesielski2@unilodz.eu (O.C.); marta.biesiekierska@unilodz.eu (M.B.); 2 The Bio-Med-Chem Doctoral School of the University of Lodz and Lodz Institutes of the Polish Academy of Sciences, University of Łódź, Banacha 12/16, 90-237 Lodz, Poland

**Keywords:** epigallocatechin gallate, epigenetics, histone acetylation, histone methylation, endothelial cells

## Abstract

Epigallocatechin gallate (EGCG), the main green tea polyphenol, exerts a wide variety of biological actions. Epigenetically, the catechin has been classified as a DNMTs inhibitor, however, its impact on histone modifications and chromatin structure is still poorly understood. The purpose of this study was to find the impact of EGCG on the histone posttranslational modifications machinery and chromatin remodeling in human endothelial cells of both microvascular (HMEC-1) and vein (HUVECs) origin. We analyzed the methylation and acetylation status of histones (Western blotting), as well as assessed the activity (fluorometric assay kit) and gene expression (qPCR) of the enzymes playing a prominent role in shaping the human epigenome. The performed analyses showed that EGCG increases histone acetylation (H3K9/14ac, H3ac), and methylation of both active (H3K4me3) and repressive (H3K9me3) chromatin marks. We also found that the catechin acts as an HDAC inhibitor in cellular and cell-free models. Additionally, we observed that EGCG affects chromatin architecture by reducing the expression of heterochromatin binding proteins: HP1α, HP1γ. Our results indicate that EGCG promotes chromatin relaxation in human endothelial cells and presents a broad epigenetic potential affecting expression and activity of epigenome modulators including HDAC5 and 7, p300, CREBP, LSD1 or KMT2A.

## 1. Introduction

Years of studies on natural compounds, i.e., sulphorafan, curcumin or ellagic acid, have revealed their potential for the development of more effective strategies for cancer prevention, support of organism regeneration after destructive cancer therapies, as well as the ability for the prevention and treatment of cardiovascular disorders [1,2,3,4]. Recently, more interest has been also given to green tea polyphenols (GTP), especially epigallocatechin-3-gallate (EGCG), that constitutes up to 45% of GTP count [5]. Growing evidence suggests that EGCG acts as a powerful antioxidant [6], inducing apoptosis and promoting tumor cell growth arrest by altering the expression of cell cycle regulatory proteins, activating caspases and suppressing NFκB transcriptional factor activation [7,8]. EGCG activates/inhibits several signaling pathways mainly by direct interaction with specific protein targets, including: (i) secreted proteases such as MMPs, (ii) membranes receptors, (iii) membrane microdomains, and (iv) the plasma membrane itself [8]. One of the first identified direct targets of EGCG was laminin receptor 67 (67LR) and later on other interacting partners, such as peptidyl-prolyl *cis/trans* isomerase 1 (Pin1) and transforming growth factor β receptor II (TGFR-II) [9,10,11,12].

The effects of EGCG on cellular metabolism are also a consequence of its epigenetic properties. EGCG has been identified as an inhibitor of DNA methyltransferases (DNMTs) that efficiently modifies DNA methylation profile [13]. In silico analyses have shown that EGCG forms hydrogen bonds with different residues in the catalytic pocket of DMNTs, causing enzyme inhibition. This prevents the methylation of the newly synthesized DNA strand, resulting in the reversal of the hypermethylation and the re-expression of silenced genes [14,15]. It has been also reported that EGCG affects folic acid metabolism in cells via the inhibition of dihydrofolate reductase activity (DHFR), causing suppression of both DNA and RNA synthesis and altering of DNA methylation pattern [16]. As EGCG generates hydrogen peroxide in substantial amounts in the auto-oxidative reactions, it may also cause oxidative damage; H_2_O_2_ can oxidize DNMTs and other proteins, altering their activity [15,17].

Recent data have provided some evidence that EGCG in cancer cells also influences the histone acetylation process. In skin cancer cells, EGCG-induced changes in global DNA methylation were accompanied by a decrease in histone deacetylases activity (HDACs) and consequent increase in histone 3 (H3) and 4 (H4) acetylation [18]. In ER α-negative breast cancer cells, the catechin increased histone acetylation levels, which in turn was correlated with upregulation and/or activation of histone acetyltransferases (HATs) [19]. Other findings point out that the treatment of colon cancer cells with EGCG significantly increases HATs and reduces HDACs activity, particularly HDAC1 [20]. The molecular background of the influence of EGCG on histone posttranslational modifications is poorly understood, and literature data on the subject is quite modest. One of the proposed mechanisms identified in colon cancer cells suggest that the catechin may contribute to the degradation of both DNMT1 and HDAC3 [21].

In general, because of antiproliferative, pro-apoptotic, and anti-oxidative properties of epigallocatechin-3-gallate, determined by the presence of phenolic rings and the trihydroxyl substitution pattern in its structure, this main green tea catechin is receiving much-warranted attention in cancer biology. In the present study, we analyzed the impact of EGCG on the endothelial cells epigenome i.e., histone posttranslational modifications, to shed more light on the molecular action of EGCG in non-tumor cells, but at the same time cells that are closely related to tumor growth and development due to neoangiogenesis and metastasis processes. Using two endothelial cell models, immortalized microvascular (HMEC-1) and primary vein (HUVECs), we studied the effect of EGCG on acetylation and methylation status of the core histone 3 (H3) and selected modifiers of the human epigenome, to figure out the role of green tea catechin in the regulation of chromatin conformation. The performed analysis revealed the significant epigenetic potential of epigallocatechin-3-gallate for modification of histone posttranslational machinery and in consequence the transcription process.

## 2. Results

### 2.1. Effect of Epigallocatechin-3-gallate (EGCG) on Proliferation of Endothelial Immortalized Cell Line and Primary Cells

**Figure 1:**
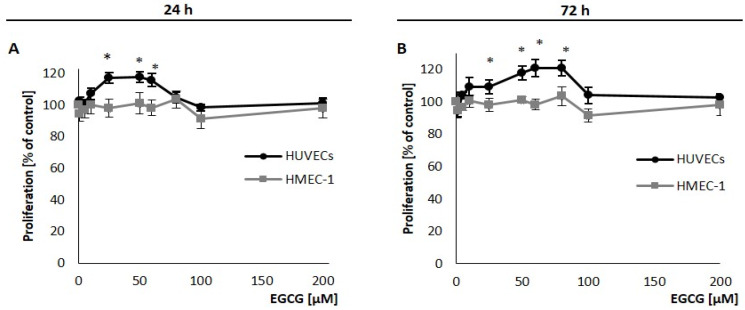
Effect of EGCG on proliferation of primary HUVECs and immortalized HMEC-1 human endothelial cells. The proliferation analysis was performed using resazurin reduction assay. Cell viability was analyzed after 24 h (**A**) and 72 h (**B**) treatment at the indicated concentrations of EGCG (for the 72 h conditions, the catechin treatment was repeated every 24 h). The data are presented as mean ± SD, *n* = 3; * *p* <0.05, control vs. treated, ANOVA and post hoc analysis by Tukey’s test.

To assess the biological effect of EGCG on human endothelial cells we analyzed its influence on the proliferation of primary HUVECs and immortalized HMEC-1 using resazurin reduction assay. The cells were treated with EGCG for 24 h or 72 h (with the compound treatment repeated every 24 h) at the 5–200 μM concentration range (Figure 1A,B). We found that EGCG has no cytotoxic effects on both endothelial cell types. Statistically significant stimulation of proliferation was observed only in primary HUVECs, at the range of concentrations 25–50 μM for both 24 h (Figure 1A) and 72 h (Figure 1B) incubation of cells with the compound. On the contrary, EGCG appears to have no effect on HMEC-1 proliferation. Neither 24 h nor 72 h combined treatment (3 × 24 h) of immortalized cells with the tested catechin affected their viability (Figure 1A,B; grey plots). Based on these results the concentration range of 25–200 μM was chosen for further studies.

### 2.2. Epigallocatechin Gallate Alters Acetylation Profile of Histone Core Protein 3 (H3) via Modulation of Expression and Activity of the Major Acetylation Status Drivers

**Figure 2:**
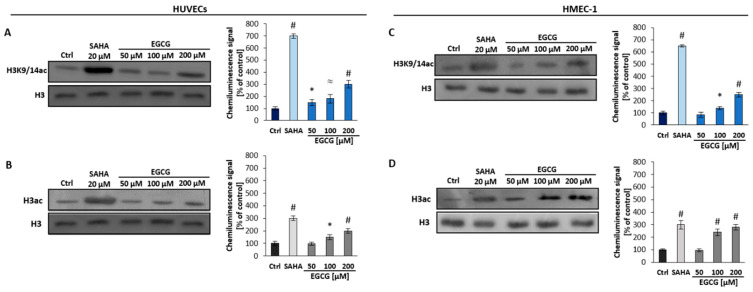
Effect of EGCG treatment on acetylation status of selected lysines (K) of the histone 3 (H3) in primary HUVECs (**A**,**B**) and immortalized HMEC-1 (**C**,**D**). Cells were incubated for 16 h with 50, 100 or 200 µM of EGCG. The proteins after acid extraction were used for the immunoblotting analysis. The presented Western blots are representative of three independent experiments and the charts visualize densitometry of the chemiluminescence signal calculated vs. loading control and vs. experiment control. The data are presented as mean ± SD; *n* = 3; * *p* < 0.05, ≈ *p* < 0.01, # *p* < 0.001, control vs. treated, ANOVA and post hoc analysis by Tukey’s test.

To elucidate the influence of EGCG on histone acetylation profile of human endothelial cells, both analyzed cell types were treated with 50, 100 or 200 μM of EGCG for 16 h. As a positive control for histone acetylation, an HDAC inhibitor–SAHA (20 μM) was used. The analysis of histone H3 acetylation profile of both pan acetyl H3 (H3ac; Figure 2B,D) and of the lysine 9/14 (H3K9/14ac; Figure 2A,C) clearly showed that the catechin treatment increases the level of modification.

**Figure 3:**
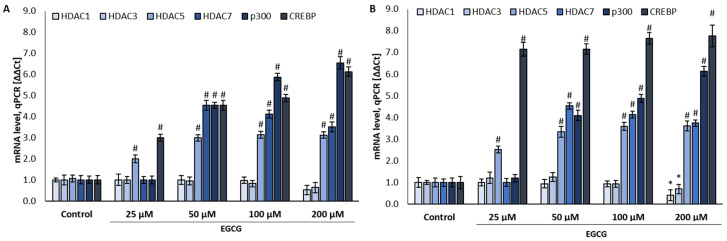
mRNA expression level of selected histone deacetylases and histone acetyltransferases in (**A**) HUVECs and (**B**) HMEC-1 after EGCG treatment. Endothelial cells were incubated with EGCG at the indicated range of concentrations for 24 h. The data are presented as mean ± SD, *n* = 3; * *p* < 0.01, # *p* < 0.01, control vs. treated, ANOVA and post hoc analysis by Tukey’s test.

Despite the observed differences in the proliferation of HUVECs and HMEC-1 upon EGCG treatment (Figure 1), the effect of EGCG on the acetylation status of endothelial cells was similar in both cell types. We found EGCG concentration-dependent increase up to almost 3-fold at 200 μM of EGCG in acetylation level of H3K9/14 in HUVECs and HMEC-1 (Figure 2A,C). Pan-acetylation of H3 was also increased in both EC types, however in HUVECs the increase was lower than in HMEC-1; 2-fold and 2.5-fold changes were observed, respectively (Figure 2B,D). As the western blotting results (Figure 2) proved promising, we investigated whether EGCG affects the expression of selected epigenetic enzymes involved in the modification of acetylation status of histones, such as acetyltransferases (HATs): p300, CREBP, and histone deacetylases (HDACs): HDAC1, HDAC3, HDAC5, and HDAC7. HUVECs and HMEC-1 were incubated with EGCG (20, 50, 100, 200 μM) for 24 h and gene expression levels were analyzed by qPCR. The obtained results show, that EGCG treatment affected the expression of both HATs and HDACs (Figure 3A,B). The expression of p300 was increased by almost 7- and 6-fold in HUVECs and HMEC-1, respectively. Similarly, the expression of mRNA for CREBP was significantly increased in both EC types, up to 8-fold in HMEC-1 (Figure 3). Contrary to our assumptions the expression of HDAC5 and 7 was also increased, however not as high as the expression of HATs, reaching as much as 2.5- to 4-fold, depending on the concentration of EGCG and analyzed cell type. HDAC1 and 3 did not respond to EGCG treatment at the concentration range of 25–100 µM, just the highest used concentration of EGCG (200 µM) significantly decreased the expression of both enzymes in HMEC-1 cell line (Figure 3B).

**Figure 4:**
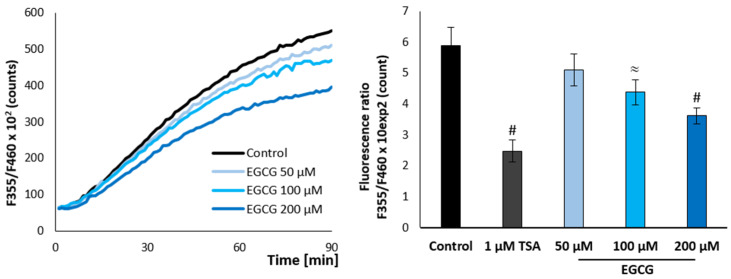
Effect of EGCG on histone deacetylase activity in a cell-free experimental model. As a positive control, an inhibitor of HDACs - 1 µM of trichostatin A (TSA) was included. HDAC activity was measured by 90 min (left chart) and fluorescence of the probe (7-amino-4-methylcoumarin, AMC) at a 90 min was used for assessment of HDAC activity (right chart), according with the manufacturer’s suggestions. The data are presented as mean ± SD, *n* = 3; ≈ *p* < 0.01, # *p* < 0.01, control vs. treated, ANOVA and post hoc analysis by Tukey’s test.

To understand the molecular background of EGCG action on histone acetylation, we assessed its inhibitory potential against HDACs with the fluorometric HDAC activity assay kit (Abcam). The analysis of HDAC activity in a cell-free experimental model showed inhibition of the enzymatic activity of deacetylases by EGCG in a dose-dependent manner (Figure 4).

**Figure 5:**
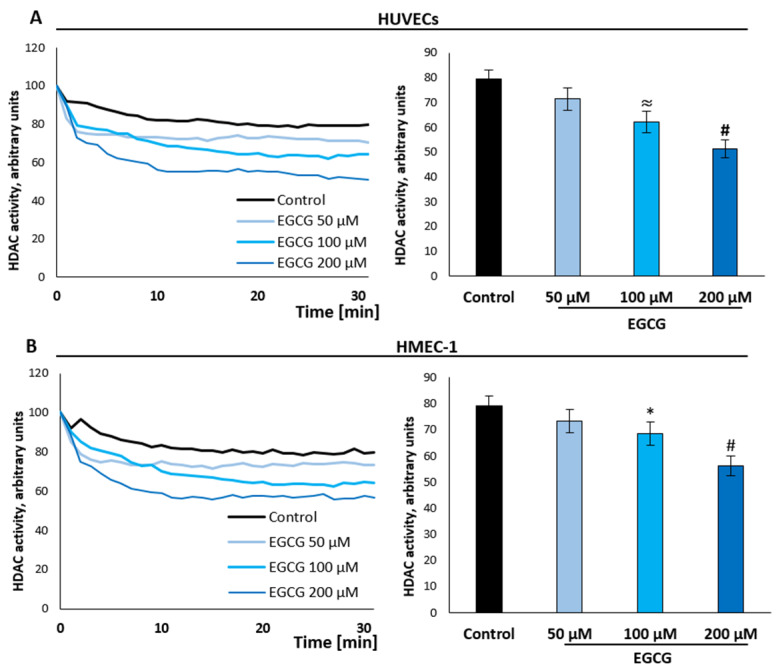
Effect of EGCG on histone deacetylase activity in (**A**) HUVECs, (**B**) HMEC-1. Cell were incubated by 16 h with 50, 100 or 200 µM of EGCG. For measurements of HDAC activity 20 µg of total cell extracts was used. The left panels present the kinetics of the reaction and the right one HDAC activity calculated according with the manufacturer’s guidelines. The data are presented as mean ± SD, *n* = 3; * *p* < 0.05, ≈ *p* < 0.01, # *p* < 0.01, control vs. treated, ANOVA and post hoc analysis by Tukey’s test.

These results were further proved by performing HDAC activity assay in endothelial cell lysates. Cells were incubated for 16 h with 50, 100 or 200 µM EGCG and then the cellular lysates were used for the estimation of the HDAC activity. Likewise in a cell-free experimental model, we observed an inhibition of HDAC activity in both endothelial cell types used, in the EGCG concentration-dependent manner (Figure 5A,B).

### 2.3. Effect of Epigallocatechin Gallate on the Methylation Profile of Histone Core Protein 3 (H3) and Gene Expression of the Selected Methylation Status Drivers

**Figure 6:**
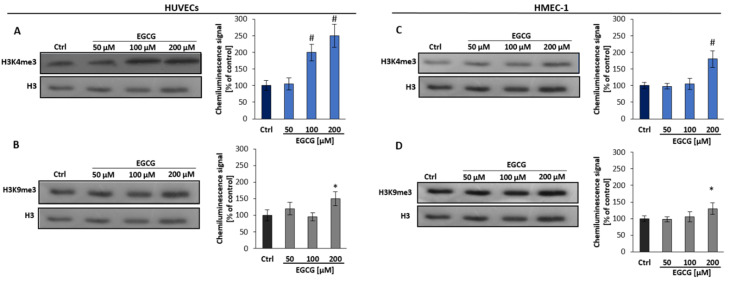
Effect of EGCG treatment on methylation status of selected lysines (K) of the histone 3 (H3) in primary HUVECs (**A**,**B**) and immortalized HMEC-1 (**C**,**D**). Cells were incubated for 16 h with 50, 100 or 200 µM of EGCG. The proteins after acid extraction were used for the immunoblotting analysis. The presented western blots are representative of three independent experiments and the charts visualise densitometry of the chemiluminescence signal calculated vs. loading control and vs. experiment control. The data are presented as mean ± SD; *n* = 3; * *p* < 0.05, # *p* < 0.01, control vs. treated, ANOVA and post hoc analysis by Tukey’s test.

Finding an increase in the acetylation status of histones due to EGCG treatment, the modification that is linked with chromatin relaxation and elevated gene expression level, we focused on the methylation process and the signatures of transcriptionally active and silent chromatin, H3K4me3 and H3K9me3, respectively. In the performed western blot analysis the aforementioned modifications showed a significant increase (almost 2.5-fold) of trimethylation of lysine 4 (H3K4me3) especially in HUVECs (Figure 6A). The repressive signature of transcription, H3K9me3, showed a slight increase up to 130–160% vs. control, both in HUVECs as well as HMEC-1, but only upon 200 μM EGCG treatment (Figure 6B,D).

**Figure 7:**
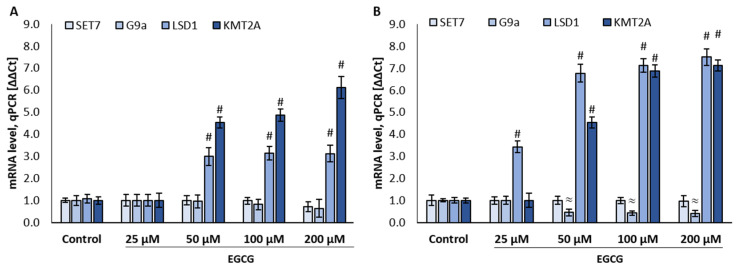
mRNA expression level of selected histone 3 methylation enzymatic drivers in (**A**) HUVECs and (**B**) HMEC-1 after EGCG treatment. Endothelial cells were incubated with EGCG at the indicated range of concentrations for 24 h. The data are presented as mean ± SD, *n* = 3; ≈ *p* < 0.01, # *p* < 0.01, control vs. treated, ANOVA and post hoc analysis by Tukey’s test.

In the next step, we looked at the expression of histone methylation associated enzymes, i.e., methyltransferases: KMT2A, G9a, SET7/9 and also lysine-specific demethylase 1 (LSD1). Except for SET7/9 methyltransferase all other analyzed genes were modified due to the EGCG treatment (Figure 7A,B). HMEC-1 responded to the catechin significantly stronger than HUVECs. We found an almost 8-fold expression increase of LSD1 and KMT2A in the EGCG concentration-dependent manner and a significant decrease of G9a expression (Figure 7B). In HUVECs we observed increased expression of KMT2A, comparable to HMEC-1 and also LSD1, about 3-fold, remained at the same level despite the increasing concentration of EGCG from 50 to 200 µM (Figure 7B).

### 2.4. Effect of Long-Term Incubation of Cells with Epigallocatechin Gallate on the Selected Histone Modification Signatures

Based on the identified changes in histone acetylation and methylation status of endothelial cells due to a single 50 µM EGCG treatment (up to 200 µM), we checked the effect of multiple treatments on selected epigenetic signatures while testing the lower catechin concentrations.

**Figure 8:**
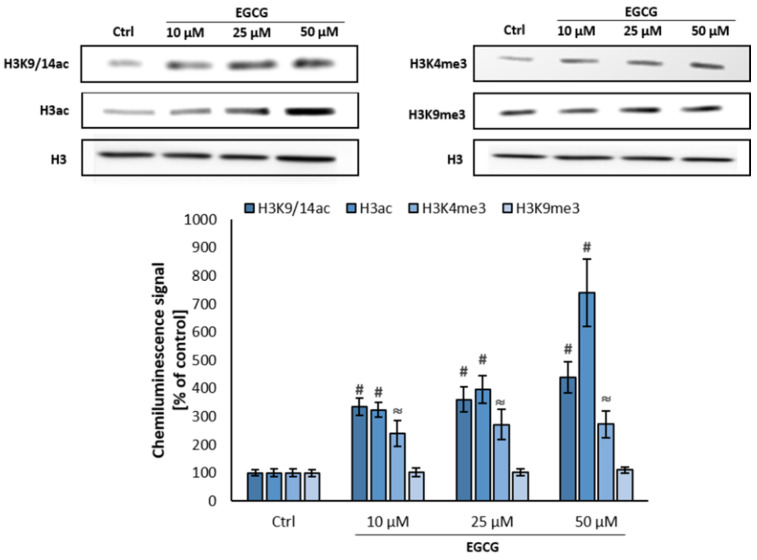
Effect of multiple EGCG treatment on acetylation and methylation profile of selected lysine residues in HMEC-1. The cells we treated with the green tea polyphenol every 24 h for 5 days. Whole cell extracts were used for the immunoblotting analysis. The presented western blots are representative of three independent experiments and the chart visualizes densitometry of the chemiluminescence signal calculated vs. loading control and vs. control. The data are presented as mean ± SD; *n* = 3; ≈ *p*< 0.01, # *p* < 0.01, control vs. treated, ANOVA and post hoc analysis by Tukey’s test.

We found that HMEC-1 treatment every 24 h for 5 days with EGCG: 10 µM, 25 µM, and 50 µM, affects histone acetylation/methylation status of microvascular endothelial cells in the same way as single exposure of cells to higher EGCG concentrations (Figure 8 vs. Figure 2C,D and Figure 6C,D). We observed a significant increase in H3K9/K14 acetylation and even stronger changes were identified at the global acetylation level of H3 (H3ac), up to 8-fold compared to control (Figure 8). Also, trimethylation of H3K4 level increased due to 5-day EGCG treatment over the entire range of concentrations used.

### 2.5. Epigallocatechin Gallate Induces Changes in Expression of Chromatin Architecture Determinants: Heterochromatin Protein 1 (HP1) and Chromatin Assembly Factor 1A (CAF1A)

Due to the identified disparities in the posttranslational modifications of histone lysine residues level within the acetylation and methylation area induced by epigallocatechin-3-gallate, we focused on the chromatin architecture. 

**Figure 9:**
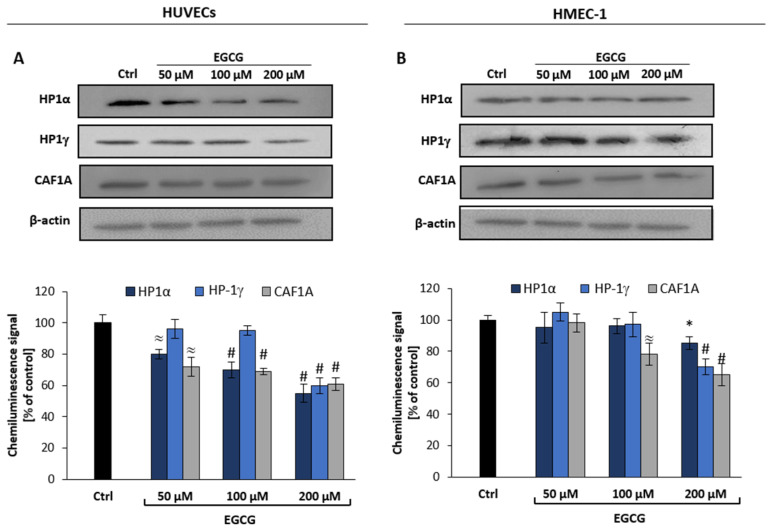
Effect of EGCG treatment on expression levels of major chromatin conformation determinants: HP-1α, HP-1γ and CAF1A in primary HUVECs (**A**) and immortalized HMEC-1 (**B**). Cells were incubated for 16 h with 50, 100 or 200 µM of EGCG. The whole cell extracts were used for the immunoblotting analysis. The presented western blots are representative of three independent experiments and the charts visualize densitometry of the chemiluminescence signal calculated vs. loading control and vs. control. The data are presented as mean ± SD; *n* = 3; * *p* < 0.05, ≈ *p* < 0.01, # *p* < 0.01, control vs. treated, ANOVA and post hoc analysis by Tukey’s test.

As before, both EC types were treated with 50, 100 or 200 μM of EGCG for 16 h and then selected parameters characterizing chromatin relaxation state were analyzed, i.e., HP-1 α/γ isoforms and chromatin assembly factor 1A (CAF1A). The obtained results showed that EGCG decreases the protein expression of heterochromatin forming proteins, specifically HP-1α in HUVEC cells, where we found EGCG concentration-dependent effect (Figure 9A). In HMEC-1 the decreased expression of HP-1α up to 80–90% of control was found only at the highest concentration of the catechin at 200 µM (Figure 9B). Similar results were obtained for HP-1γ. Only 200 µM concentration of EGCG affected HP-1γ protein expression, up to 60% in HUVECs, and up to 70% in HMEC-1 (Figure 9A,B). Also, expression of the chromatin assembly factor 1A (CAF1A) was significantly downregulated at the protein level by EGCG, (Figure 9A,B), in HUVECs even 50 µM EGCG decreased the expression of CAF1A, whereas in HMEC-1 statistically significant downregulation of CAF1A was detected after treatment of cells with 100 µM EGCG.

## 3. Discussion

Recent years of studies admittedly show that epigenetic mechanisms, as DNA methylation, histone posttranslational modifications or miRNA, play one of the key roles in defining and regulating cellular metabolism due to affecting chromatin architecture and thus transcription processes [22,23,24,25,26]. Some of these modifications are associated with pathological states, for instance in tumors a specific pattern of DNA methylation can be observed [27] and histone acetylation was found as crucial in the development of Huntington’s disease [28]. Multiple research has shown the importance of dietary habits and the impact of various compounds of natural origin in the prevention and support of the treatment of many diseases, including cancer [29,30].

Green tea catechin, EGCG, was previously mainly shown as an efficient inhibitor of DNMTs in human cancer cells [30], however, its effect on histone modifications and chromatin functions is still not fully identified, which is what we focused on in this research. As an experimental model, we used endothelial cells, closely related to cancer biology, as newly formed blood vessels work as nutrients/oxygen supply pipelines for the growing tumor. We found that EGCG stimulates only proliferation of primary endothelial cells (HUVECs), while it has no effect on immortalized cells (HMEC-1), Figure 1, which are the kind of the equivalent of cancer cells in vitro. Interestingly, when it comes to cancer cells, literature data show that EGCG inhibits their proliferation and induces apoptosis process [31]. Cytotoxic effects of EGCG on cancer cells (lung, pancreatic or colon) have been observed above 10 or 20 µM of EGCG, depending on the cell line tested [31,32,33]. Analysis of the migration process in normal endothelial cells (NECs) and tumor endothelial cells (TECs) in response to EGCG treatment also revealed significant changes between both cell types. It has been observed that 25 and 50 µM of EGCG significantly suppresses the migration of TECs (oral carcinoma and melanoma cancer endothelial cells) but not NECs [34]. The differentiated effect of EGCG on cell proliferation depending on their type (primary, immortalized or cancer) shows the multithreaded activity of the studied catechin and also enhances the legitimacy of its application in anticancer therapy.

Our further analysis showed that the differences identified in proliferation between immortalized and primary endothelial cells as a result of EGCG treatment are not applicable to the overall epigenetic response of ECs to the tested catechin. We focused particularly on histone posttranslational modifications exerting the biggest influence on chromatin architecture. In both analyzed EC types, we found a significantly increased level of acetylation. Although the concentrations of EGCG for the single treatment were quite high (in the range of 50–200 µM), we obtained coherent data for the multiple treatments (5 days) with 10, 25 and 50 µM of the catechin (H3K9/14ac and total H3ac; Figure 2 and Figure 8). These data are in line with the effects caused by EGCG (5–100 µM) on core histones acetylation identified in cancer cells, including skin, breast, and colon cancer cell lines [18,19,20]. Overacetylation of histone 3 in HUVECs and HMEC-1 was accompanied by a decreased activity of histone deacetylases (Figure 4 and Figure 5). The data obtained in the fluorometric reaction in the cell-free system show that EGCG works as an HDAC inhibitor, which was confirmed in cellular experimental models: non-tumor (Figure 5) as well as tumor cell lines, e.g., colon carcinoma cell line HT29 [20]. This again strengthens the anticancer potential of EGCG, as approved HDAC inhibitors, vorinostat, panobinostat, belinostat or romidepsin, are successfully used in cancer therapies. The subsequent studies, in-depth about the transcriptome of several histone acetylation drivers (Figure 3), revealed significantly increased HATs expression level, CREBP, and p300, but also the expression of several HDACs especially 5 and 7 (however, below the HATs range level). HDAC1 and HDAC3 did not react to EGCG treatment in the primary cells, whereas the expression of both enzymes was decreased in the immortalized cell line. The HDACs, that expression was analyzed in the presented study, were chosen based on the literature findings showing their key role in the regulation of major endothelial cell functions [35,36,37,38,39]. We found that despite the common direction of changes in the acetylation level of core histone proteins in cancer and non-cancer cells, the molecular picture of the expression/activity of the acetylation process regulators significantly differ between the cell types. Similarly to HMEC-1, in colon cancer cells downregulation of HDAC1 and HDAC3 in response to EGCG was also found [20,21], but in lung cancer cells downregulation of HDAC4, 5 and 6 was observed upon the co-treatment with EGCG and synthetic retinoid Am80 [40], whereas in the prostate cancer cells increased level of histone acetylation was correlated with HATs inhibition [41], contrary to the results obtained in the presented study where upregulation of CREBP and p300 was found.

The impact of EGCG on histone methylation has so far been analyzed from the level of cancer cells and polycomb group (PcG) protein, particularly EZH2, but also Suz12, Mel18 and Bmi-1 [18]. It was found that in skin cancer cell lines (SCC-13 and A431 cells) and prostate cancer cells (DUPRO and LNCaP) EGCG suppresses activity/expression of PcG protein and reduces the level of H3K27me3, a histone modification associated with closed chromatin and considered as a hallmark of gene silencing [18,42]. Here, the analysis of effects of EGCG on histone methylation status together with changes in acetylation, further proved that EGCG acts on ECs as a chromatin relaxing compound. Significant alterations were observed especially in the level of active chromatin marker–H3K4me3 with slight changes in silent chromatin marker H3K9me3 in primary HUVECs (Figure 4), confirmed also by multiple-dose treatments (Figure 8). Analysis of gene expression of proteins involved in methylation of lysine 4 and 9 of histone 3 revealed a significantly increased level of KMT2A that may explain the changes in H3K4me3 upon EGCG treatment [43], but also the expression of LSD1 was altered. LSD1 is reported to demethylate both H3K4me1/me2 and H3K9 me1/me2, and in many types of cancer its overexpression was proven to correlate with the poor prognosis [44,45]. This made the demethylase a target molecule of the ongoing clinical trials on cancer [27,45]. It is difficult to explain the increase in LSD1 expression due to EGCG that shows up as a supporting molecule in anticancer therapy, but the role of LSD1 in non-tumor cells may differ from tumor one, as we proved recently investigating the effect of LSD1 silencing on cell cycle of ECs [46] and also, there is no data about participation of LSD1 in reprogramming of normal to tumour endothelial cells (NECs to TECs).

The performed analysis of core histone modifications showed that EGCG acts as a chromatin relaxing agent in ECs, that was further validated by the expression levels of heterochromatin associated proteins HP1α, HP1γ and the chromatin assembly factor CAF1A. The isoform α of HP-1 is primarily associated with centromeric heterochromatin, while γ is associated with both heterochromatin and euchromatin. Nucleosome assembly factor (CAF1) deposits newly synthesized and acetylated histones H3 and H4 into nascent chromatin during DNA replication. Like HP-1γ, CAF1 is targeted to heterochromatic and euchromatic DNA replication foci fractions. Literature data present that a mark of heterochromatin dynamics–CAF1A, functions in a complex with HP1α and HP1γ proteins, but also newly synthesized histones H3 and H4 [47,48]. A significantly reduced expression of all analyzed parameters was observed in both EC types, however bigger changes were detected in primary cells (Figure 9).

## 4. Materials and Methods

### 4.1. Cell Culture

Human Microvascular Endothelial Cells (HMEC-1) were obtained from the American Type Culture Collection (Manassas, VA, USA). Human Umbilical Vein Endothelial Cells (HUVECs) were isolated from the veins of umbilical cords, by collagenase type II digestion as described previously [49]. Both endothelial cell types were cultured in MCDB131 medium (Corning Life Sciences, Corning, NY, USA), supplemented with 10% foetal bovine serum (EurX, Gdańsk, Poland), 10 ng/mL Epidermal Growth Factor (Millipore, Burlington, MA, USA) and 10 mM glutamine (Corning Life Sciences).

### 4.2. Cell Proliferation Analysis by Resazurin Reduction Assay

Cell proliferation was analyzed using the ability of live cells to reduce resazurin to fluorescent resorufin, as it was described previously [50]. Both endothelial cell types were seeded onto 96-well plates at a density of 5000 and 7000, respectively for 24 h and 72 h incubation. After 16–20 h the cells were treated with EGCG (Sigma-Aldrich, Saint Louis, Missouri, USA) at the concentration range of 5–200 μM for 24 h or 72 h with a medium change every 24 h. After the time indicated above the culture medium was removed, cells were washed with PBS containing Ca^2+^/Mg^2+^ and 5.5 mM glucose. Then the cells were incubated for 2 h in PBS containing Ca^2+^/Mg^2+^, 5.5 mM glucose with 0.0125 mg/mL resazurin. After incubation fluorescence was read on a Fluoroscan Ascent microplate reader (Thermo-Fisher Scientific, Waltham, MA, USA) at λ_ex_ = 530 nm, λ_em_ = 590 nm (Table 1).

### 4.3. RNA Isolation, Reverse Transcription and Real-Time PCR

Cells were plated onto 6-well plates and after 16–20 h treated with 25, 50, 100 or 200 μM EGCG for 24 h. Total cellular RNA was isolated and purified using InviTrap^®^Spin Cell RNA mini kit (Stratec Molecular, Birkenfeld, Germany), in accordance with the manufacturer’s protocol. cDNA synthesis was performed with PrimeScriptTM RT Reagent Kit (Perfect Real Time, Takara, Japan), according to instructions provided with the kit. Real-Time PCR was performed on Eco Real-Time PCR (Illumina; San Diego, CA, USA). The final reaction volume was 10 μL and contained: 0.2 nM of forward and reverse primers, cDNA template, SYBR Green (Perfect Real Time, Takara) and DNAase/RNAase free water. Reactions were incubated at 96 °C for 2 min, followed by 40 cycles of 96 °C for 5 s and 60 °C for 30 s. HPRT1 was used as a reference for gene expression normalization, performed according to the 2^− ΔΔCt^ method [51].

### 4.4. Western Blotting

Cells were seeded onto 60 mm dishes at 3,500,000 cells/dish and after 18–20 h treated with EGCG (50, 100 or 200 μM), and incubated with the catechin for 16 h. Histone extracts were prepared following the acid extraction protocol as described previously [52], whereas the whole-cell extracts were prepared using M-PER (Thermo-Fisher Scientific, Waltham, MA, USA) following the manufacturer’s protocol. The extracts were immunoblotted to determine the pattern of acetylation/methylation of lysine residues of histone H3 (acid extracts) and the expression levels of HP1α, HP1γ, and CAF1A (whole-cell extract). One µg of histone extract or 5 µg whole-cell extract was loaded onto polyacrylamide gels and resolved using SDS-PAGE, then transferred to PVDF. The membrane was washed with TBST buffer (10 mM Tris, pH 8.0, 150 mM NaCl, 0.5% Tween20) and blocked in 3% freshly prepared non-fat milk in TBST overnight at 4 °C with agitation. After incubation, the membrane was washed three times for 5 min in TBST at room temperature. Next, the membranes were probed with primary antibodies: H3ac (cat. no. ab47915, Abcam, Cambridge, UK), H3K9/14ac (cat. no. C15410005, Diagenode, Liège, Belgium), H3K4me3 (cat. no. #39159, Active Motive, La Hulpe, Belgium), H3K9me3 (cat. no. #39161, Active Motive, La Hulpe, Belgium); H3 (cat. no. ab18521, Abcam, Cambridge, United-Kingdom) was used as a loading control. Additionally, HP1α (cat. no. #C1019, Cell Signalling, Leiden, The Netherlands), HP1γ (cat. no. #C1016, Cell Signalling, Leiden, The Netherlands) and CAF1A (cat. no. #C5480, Cell Signalling, Leiden, The Netherlands ) were used with β-actin (mAbcam 8226, Abcam, Cambridge, United-Kingdom) as a loading control. The membranes were incubated with the primary antibodies for 1.5 h (histones) or overnight (other analyzed antigens) and then washed three times in TBST for 5 min. Next, the membranes were incubated with horseradish peroxidase-conjugated secondary antibodies (1:2000, cat. no. #HAF007, cat. no. #HAF008, R&D Systems, Minneapolis, MN, USA) for 1.5 h at room temperature. The signal from the membranes was visualized using ECL (WESTAR ETA C 2.0, Cyanagen, Bologna, Italy). Densitometry was normalized to the loading control (unmodified histone H3 or β-actin) and then to the experiment control.

### 4.5. Histone Deacetylase Activity Assay

The HDAC inhibitory potential of EGCG was measured in vitro, in both endothelial cells models. The analysis was performed using the HDAC activity fluorometric assay kit (cat# ab156064, Abcam) as described previously [53], following the manufacturer’s protocol. Briefly, for in vitro HDAC activity assay ECs were plated onto 60 mm dishes and treated with EGCG (50, 100 or 200 μM) for 16 h. Ten cellular lysates were prepared using the M-PER kit. The ability of EGCG to act as an HDAC inhibitor (HDACi) was also measured in a cell-free experimental model, where the inhibitory action of the tested compound was compared with the trichostatin A (TSA) a well-known HDACi.

### 4.6. Statistical Analysis

Statistical analyses were performed by means of STATISTICA 8.0 PL software (StatSoft INC, Tulsa, OK, USA). All data were expressed as mean ± SD. Differences between groups were assessed by the one-way ANOVA and post hoc analysis by Tukey’s test was performed. A statistical significance was analyzed at *p* < 0.05, *p* < 0.01 and *p* < 0.001.

## 5. Conclusions

**Scheme 1:**
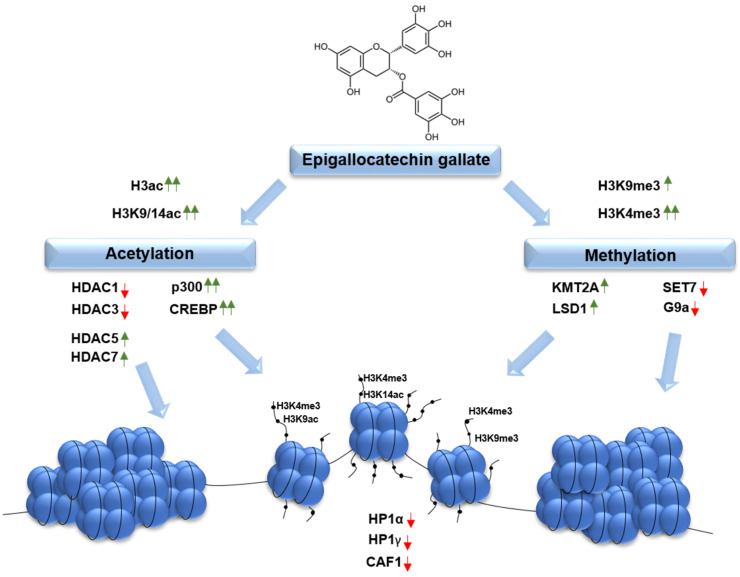
Summary of the epigenetics effects exerted by epigallocatchin-3-gallate, visualizing its potential for modification of chromatin architecture via changes in acetylation and methylation of histone proteins and DNA methylation pattern. The scheme does not include the potential crosstalk between the epigenetic modifications, however, chromatin architecture can be modified not only by the direct effect of EGCG on histone marks but also indirectly as a result of inhibition of DNMTs activity by the catchin. (HP1—heterochromatin protein 1; CAF1A—nucleosome assembly factor, subunit p150; HDAC—histone deacetylase; p300—histone acetyltransferase; CREBP—CREB-binding protein; LSD1—lysine specific demethylase 1; KMT2A—lysine methyltransferase 2A, G9a—lysine methyltransferase; CpG—or CpG island, regions of DNA rich in a cytosine followed by a guanine nucleotide).

Taken together, as summarized on Scheme 1, the epigenetic potential of EGCG is not only limited to DNA methylation, but the molecule also modulates the chromatin architecture through histone posttranslational modifications, affecting also the expression of acetylation/methylation driver enzymes and thus heterochromatin formation. The studies also show that the effect of EGCG is varied between the cell types, especially non-tumor versus tumor, supporting the role of the compound in cancer treatment.

## Figures and Tables

**Table 1:** Primer sequences used in the study for gene expression analysis.

Gene	Primer Sequence
*HPRT1*	F–TCCATTCCTATGACTGTAGATTT R–AACTTTTATGTCCCCCGTTGATT
*HDAC1*	F–ACTGGTGGTCTGTGTTCTGTGA R–GATGCCAGTCTTACTCATAGCTAC
*HDAC3*	F–TGGTGAATGGACACCAACTC R–TAGCATGCTTCGATGTGGCA
*HDAC5*	F–ATGCCAACCTCCTCAACGACC R–TCTGTTCCTCGCAGACCTCCA
*HDAC7*	F–GCCTGTACTGAGCTGGGCAAA R–TTTTGGCTGCAGAGAGGTGCA
*CREBP*	F–ATGCCAACCTCCTCAACGACC R–TCTGTTCCTCGCAGACCTCCA
*p300*	F–CCAGACCAGCATGACAGATTTC R–GCTTCCTCTTGGAGCAGATCAG
*G9a*	R–TGGGGCATTGATTGCATCTGG F–TCTCAACTGAAGCTCGCACT
*KMT2A*	F–ACTGGTGGTCTGTGTTCTGTGA R–GATGCCAGTCTTACTCATAGCTAC
*SET7*	F–ATGCCAACCTCCTCAACGACC R–TCTGTTCCTCGCAGACCTCCA
*LSD1*	F–ATGCCAACCTCCTCAACGACC R–TCTGTTCCTCGCAGACCTCCA
